# Enzymatic Synthesis of Fatty Hydroxamic Acid Derivatives Based on Palm Kernel Oil

**DOI:** 10.3390/molecules16086634

**Published:** 2011-08-05

**Authors:** Hossein Jahangirian, Md Jelas Haron, Nor Azah Yusof, Sidik Silong, Anuar Kassim, Roshanak Rafiee-Moghaddam, Mazyar Peyda, Yadollah Gharayebi

**Affiliations:** 1 Department of Chemistry, Faculty of Science, Universiti Putra Malaysia, UPM Serdang, Selangor 43400, Malaysia; 2 School of Chemical Sciences and food Technology, Faculty of science and Technology, Universiti Kebangsaan Malaysia, UKM Bangi, Selangor 43600, Malaysia; 3 Department of Chemical and Environmental Engineering, Faculty of Engineering, Universiti Putra Malaysia, UPM Serdang, Selangor 43400, Malaysia

**Keywords:** benzyl fatty hydroxamic acids, methyl fatty hydroxamic acids, isopropyl fatty hydroxamic acids, enzymatic reaction, palm kernel oil, lipozyme

## Abstract

Fatty hydroxamic acid derivatives were synthesized using Lipozyme TL IM catalyst at biphasic medium as the palm kernel oil was dissolved in hexane and hydroxylamine derivatives were dissolved in water: (1) *N*-methyl fatty hydroxamic acids (MFHAs); (2) *N*-isopropyl fatty hydroxamic acids (IPFHAs) and (3) *N*-benzyl fatty hydroxamic acids (BFHAs) were synthesized by reaction of palm kernel oil and *N*-methyl hydroxylamine (*N*-MHA), *N*-isopropyl hydroxylamine (*N*-IPHA) and *N*-benzyl hydroxylamine (*N*-BHA), respectively. Finally, after separation the products were characterized by color testing, elemental analysis, FT-IR and ^1^H-NMR spectroscopy. For achieving the highest conversion percentage of product the optimum molar ratio of reactants was obtained by changing the ratio of reactants while other reaction parameters were kept constant. For synthesis of MFHAs the optimum mol ratio of *N*-MHA/palm kernel oil = 6/1 and the highest conversion was 77.8%, for synthesis of IPFHAs the optimum mol ratio of *N*-IPHA/palm kernel oil = 7/1 and the highest conversion was 65.4% and for synthesis of BFHAs the optimum mol ratio of *N*-BHA/palm kernel oil = 7/1 and the highest conversion was 61.7%.

## 1. Introduction

Hydroxamic acids and their derivatives with the general formula R–CO–NHOH and R–CO–NR′OH have wide applications due to their biological activities and chelating properties. These compounds are weak organic acids with low toxicity. In the recent half century, they have been widely investigated for a variety of applications such as antibacterial agents, fungicide agents, growth factors, neurotoxic drug for anti HIV drugs, antimalarial drugs, enzyme inhibitors, cell-division factors, rare earth mineral collectors, and reagents for solvent extraction and spectrophotometric determination of metals [1,2,3,4,5,6,7,8,9,10].

Hydroxamic acids were generally synthesized by the chemical reaction of alkyl and aryl esters or activated carboxylic acids with hydroxylamine or its derivatives in a highly alkaline medium [11,12,13]. These reactions were usually carried out under severe conditions and involve many steps so they are often expensive processes. Accordingly, many researchers have applied enzymatic reactions for synthesis of hydroxamic acids due to their mild reaction conditions. Biotechnological synthesis of a new class of fatty hydroxamic acids carried out using the lipase of *Mucor miehei* by reacting hydroxylamine with the fatty acids in their free or methyl ester form was reported by Servat *et al.* [14]. They studied different parameters to determine the optimum reaction conditions and finally described a general method for any type of fatty acids used. The use of lipase as a biocatalyst was improved by immobilizing it on Duolite A-378 resin for the production of short-chain hydroxamic acids such as acrylohydroxamic acid and propionohydroxamic acid and production of medium chain hydroxamic acids such as butyrohydroxamic acid, valerohydroxamic acid, and several *o*-aminohydroxamic acid [15]. The researchers then improved their work and described the enzymatic synthesis of monohydroxamic acids with various chain lengths (C_2_ to C_18_) using three microbial enzymes (lipase-acyl transferase from *Candida parapsilosis*, adipamidase and wide spectrum amidase from *Rhodococcus sp. R312*). They investigated the effect of pH, temperature and mole ratio of the reactants on the reaction and obtained mild conditions such as pH, 7 to 8 and temperature below 50 °C [16].

Fatty hydroxamic acids were synthesised from fatty acids or fatty acid methyl esters and hydroxylamine by using lipase-acyltransferase from *Candida Parapsilopsis* as catalyst in a biphasic lipid/aqueous medium by Vaysse *et al.* [17]. Recently the synthesis of fatty hydroxamic acids (FHAs) from canola oil and hydroxylamine has been reported by our group [18]. In this investigation the reaction was carried out in biphasic medium in the present of lipozyme as immobilized enzyme, for optimization of the reaction we also investigated the effect of changing of reaction conditions such as types of organic solvent, pH, amount of enzyme, mol ratio of reactants and reaction time on the reaction. In the latest report, we have investigated the synthesis of the phenyl fatty hydroxamic acids (PFHAs) as the first derivative of fatty hydroxamic acids [19]. In this synthesis, phenyl hydroxylaminolysis of canola or palm kernel oils were carried out in biphasic organic/aqueous medium using lipozyme as catalyst. The oils used in this synthesis had different compositions of saturated and unsaturated natural fatty acids with 12 to 22 carbon atoms in the aliphatic chain. Hence, the obtained procedure is also applicable to other vegetable oils.

In the present investigation, we carried out the synthesis of MFHAs, IPFHAs and BFHAs separately from *N*-MHA, *N*-IPHA or *N*-BHA, respectively, and palm kernel oil using an immobilized lipase catalyst. This is the first report concerning the synthesis of fatty hydroxamic acids derivatives based on commercial palm kernel oil. The oil is easily available and often low cost which is among the advantages of these reactions. Moreover, the reactions were catalyzed by an immobilized lipase catalysts, thus it include all the advantages of enzymatic reactions such as high selectivity, good saving in energy, mild reaction conditions and environmental friendliness.

## 2. Results and Discussion

### 2.1. Synthesis

**Scheme 1:**
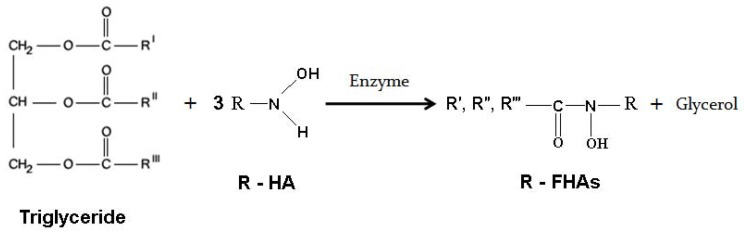
The reaction equations of methyl hydroxylaminolysis (R = methyl), isopropyl hydroxylaminolysis (R = isopropyl) and benzyl hydroxylaminolysis (R = benzyl) of triglycerides. Enzyme = Lipozyme.

Scheme 1 shows the general equations of methyl-, isopropyl- and benzyl hydroxylaminolysis of the triglyceride (palm kernel oil). Since the nature of the present reactions are similar to that of phenyl hydroxylaminolysis of canola oil (a triglyceride) published recently by our group [19] we used similar optimized conditions such as type of organic solvent, reaction period, type and amount of enzyme, and temperature in the present work as follows: organic solvent, hexane; reaction period, 72 h; type and amount of lipase, 80 mg Lipozyme TL IM/mmol oil and temperature, 39 °C.

### 2.2. Effect of Mol Ratio of Reactants

In an equilibrium chemical reaction such as an enzymatic reaction, the mol ratio of reactants is one of the most effective parameters for achieving the highest yield. Hence, in this study various mol ratios of reactants for obtaining maximum conversion were investigated. To achieve this objective, different amounts of *N*-MHA, *N*-IPHA and *N*-BHA were used in the reaction mixtures while the amount of oil and other parameters were kept constant.

**Figure 1:**
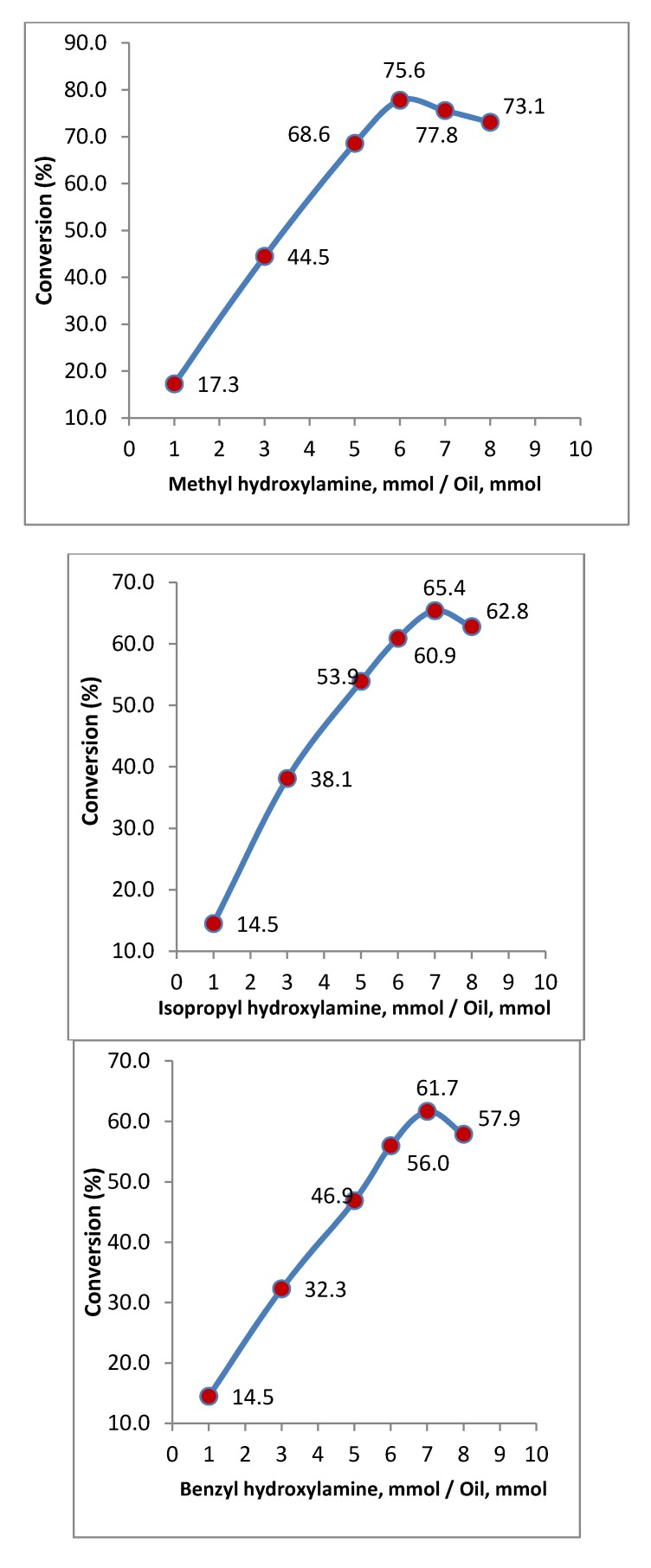
Effect of the mol ratio of *N*-MHA to oil on methyl hydroxylaminolysis, isopropyl hydroxylaminolysis and benzyl hydroxylaminolysis of palm kernel oil. Reaction conditions: Reaction time = 72 h, temperature = 39 °C, shaking rate = 120 rpm, Lipozyme TL IM = 80 mg, hexane = 10 mL, H_2_O = 14 mL, palm kernel oil = 710 mg (1 mmol).

Figure 1 shows the percentage of conversion in methyl hydroxylaminolysis, isopropyl hydroxylaminolysis and benzyl hydroxylaminolysis of palm kernel oil for various ratios of reagents. The figure shows that when mol ratio of *N*-MHA to palm kernel oil, *N*-IPHA to palm kernel oil or *N*-BPHA to palm kernel oil increases, the reaction yields increase until they reached a maximum. The maximum yields occurred at the ratios of 6 mmol of *N*-MHA, 7 mmol of *N*-IPHA or 7 mmol of *N*-BHA to 1 mmol of palm kernel oil, respectively. The steep increase in the yield before the maximum point is the result of the equilibrium shift due to an increase in the amount of *N*-MHA, *N*-IPHA or *N*-BHA in the aqueous phase. However further increases in the amount of *N*-MHA, *N*-IPHA or *N*-BHA caused the yields to decrease, which could be due to saturation of *N*-MHA, *N*-IPHA or *N*-BHA in the aqueous phase causing them to dissolve in the organic phase and inhibit the enzyme. A similar behavior was reported by Vaysse *et al.* [17] in hydroxylaminolysis of fatty acids or fatty acid methyl esters, Suhendra *et al.* [20] in hydroxylaminolysis of palm oils, Jahangirian *et al.* [18] in hydroxylaminolysis of canola oil and Jahangirian *et al.* [19] in phenylhydroxylaminolysis of canola and palm oils.

### 2.3. Characterization of MFHAs, IPFHAs and BFHAs

The complexes of the MFHAs and BFHAs separately with vanadium (V), iron (III) and copper (II) were purple, dark red and green, respectively. The complex of the IPFHAs with copper (II) was green. These are the common color of the complexes observed when these metal ions are reacted with hydroxamic acids [18,19,20].

#### 2.3.1. Elemental analysis

Results of elemental analysis showed that the nitrogen content in the MFHAs, IPFHAs and BFHAs synthesized from the palm kernel oil were 5.31, 4.62 and 3.91% respectively.

#### 2.3.2. Fourier Transform Infrared Spectroscopy (FTIR)

**Figure 2:**
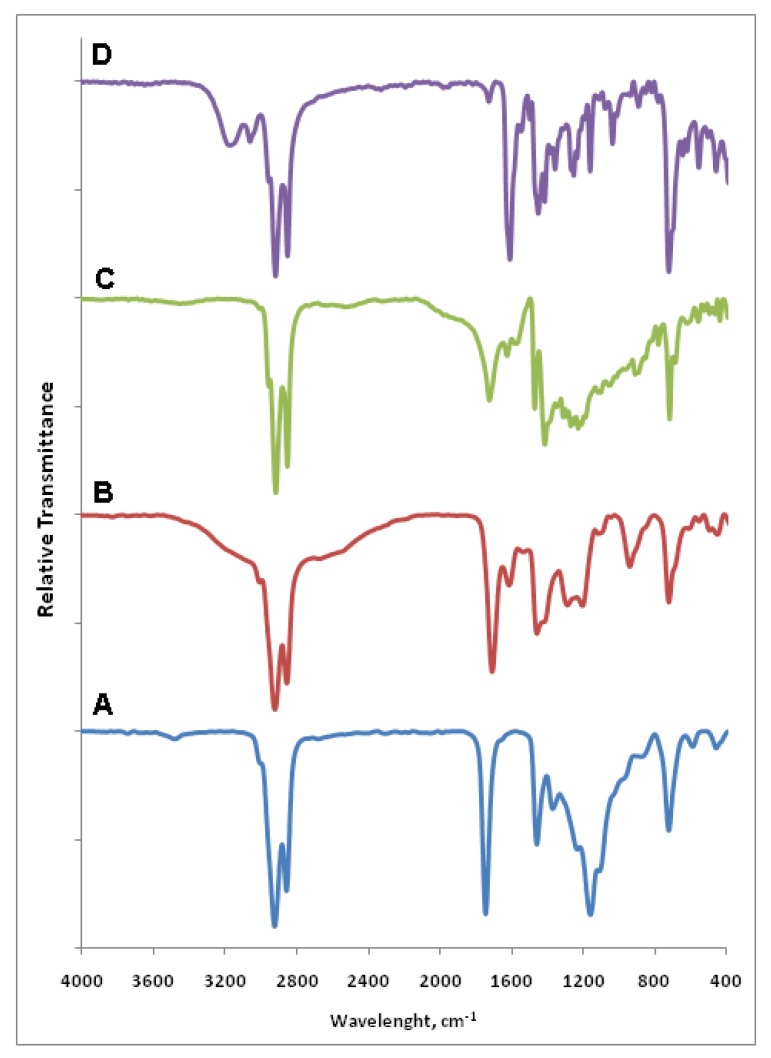
FTIR spectra of palm kernel oil (A), MFHAs (B), IPFHAs (C) and BFHAs (D).

FTIR spectra of palm kernel oil (A), MFHAs (B), IPFHAs (C) and BFHAs (D) are shown in Figure 2. In the palm kernel oil spectrum (A) the peaks at 2922 and 2855 cm^−1^ correspond to –C–H stretching for the long chain of alkyl, while the peaks at 1743, 1458 and 1158 cm^−1^ correspond to C=O, C=C and C–O stretching, respectively [21].

In the MFHAs spectra (B) the broad peaks that spread between 2700 and 3200 cm^−1^ correspond to O–H stretching, the weak peak at 3010 cm^−1^ corresponds to =C–H stretching, the peaks at 2920 and 2853 cm^−1^ correspond to –C–H stretching for the long chain of alkyl and the peak at 1707 cm^−1^ corresponds to C=O stretching that in accordance with the carbonyl peak of phenyl fatty hydroxamic acid which appeared at 1708 cm^−1^ as reported by our group recently [19]. In addition the peaks at 1612 and 1457 cm^−1^ correspond to C=C stretching, the peaks at 1286 and 1203 cm^−1^ correspond to –C–N stretching and finally the peak at 721 cm^−1^ correspond to =C–H out of plan (OOP) bending.

In the IPFHAs spectra (C) the peak of O–H stretching was disappeared due to formation of intermolecular hydrogen bonding, the peak at 2955 cm^−1^ corresponds to =C–H stretching and the peaks at 2915 and 2850 cm^−1^ correspond to –C–H stretching for the long chain alkyl. The peak at 1723 cm^−1^ corresponds to C=O stretching that has shifted to upper frequencies compare to carbonyl peak of MFHAs. In addition the peaks at 1624, 1470 and 1414 cm^−1^ correspond to C=C stretching, the peaks at 1310 and 1227 cm^−1^ correspond to –C–N stretchings, and finally the peak at 716 cm^−1^ corresponds to =C–H out of plan (OOP) bending.

In the BFHA spectra (D) the peak appearing at 3170 cm^−1^ corresponds to O–H stretching, the peaks at 3059 and 3012 cm^−1^ correspond to =C–H stretching, and when compared to =C–H peaks of MFHAs, have shifted to upper frequencies and also have more strongly appeared due to the presence of aromatic ring. The peaks at 2917 and 2849 cm^−1^ correspond to –C–H stretching for the long chain of alkyl, the peak at 1608 cm^−1^ corresponds to C=O stretching that a has shifted to lower frequencies compared to carbonyl peaks of MFHAs and IPFHAs. In addition the peaks at 1545, 1450 and 1414 cm^−1^ correspond to C=C stretching, the peaks at 1252 and 1160 cm^−1^ correspond to –C–N stretchings, and finally the peak at 721 cm^−1^ corresponds to =C–H out of plan (OOP) bending.

#### 2.3.3. ^1^H Nuclear Magnetic Resonance (^1^H-NMR)

**Figure 3:**
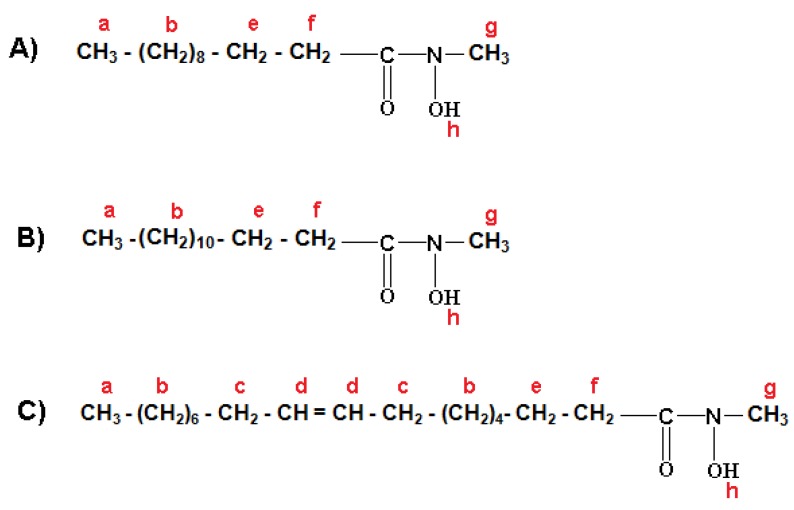
Formulation of methyl laureohydroxamic acid (A); methyl myristohydroxamic acid (B); methyl oleohydroxamic acid (C).

**Figure 4:**
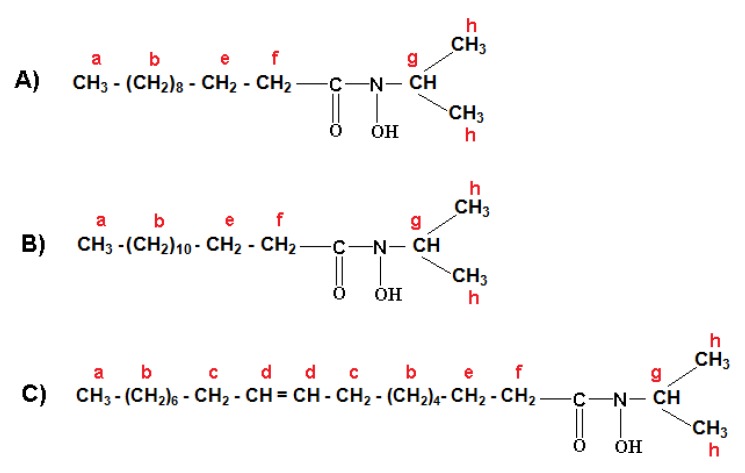
Formulation of isoprophyl laureo hydroxamic acid (A); isoprophyl myristo hydroxamic acid (B); isoprophyl oleo hydroxamic acid (C).

**Figure 5:**
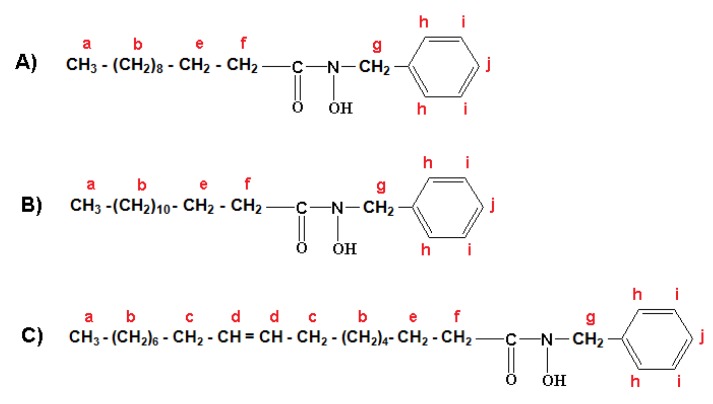
Formulation of benzyl laureo hydroxamic acid (A); benzyl myristo hydroxamic acid (B); benzyl oleo hydroxamic acid (C).

The formulation of three major fractions of MFHAs, IPFHAs and BFHAs that produced based on the palm kernel oil are shown in Figure 3, Figure 4 and Figure 5, respectively, where hydrogen atoms were labeled by a, b, c, d, e, f, g, h, i and j.

^1^H-NMR spectra of MFHAs showed signals at a (0.869, 0.881 and 0.892 ppm), b (1.254, 1.269, 1.287, 1.289, 1.300 and 1.303 ppm), c (1.996, 2.006, 2.016, and 2.027 ppm), d (5.316, 5.325, 5.340, 5.347 and 5.353 ppm), e (1.595, 1607, 1.617, 1.628 and 1.640 ppm) and f (2.309, 2.321 and 2.334 ppm) corresponding to the alkyl branch hydrogens of the MFHAs that are shown in Figure 3. These signals were confirmed for the alkyl branch hydrogens of lauric-, myristic- and oleic acids [19,22,23]. Also the signal at g (3.356 ppm) corresponds to the methyl group hydrogens beside the hydroxamic acid functional group. In addition ^1^H-NMR signals of hydrogen related to the hydroxamic acid functional group appeared at h (7.626 ppm), this signal is single, wide, weak and also it is only detectable by a NMR-600 MHz apparatus and is recognized as the main and specific signal of hydroxamic acid functional groups.

^1^H-NMR spectra of IPFHAs showed the signals at a (0.895, 0.907 and 0.917 ppm), b (1.281, 1.294 and 1.310 ppm), c (2.031, 2.041, 2.069, 2.061 and 2.092 ppm), d (5.351, 5.370, 5.372, 5.391 and 5.402 ppm), e (1.634 and 1.644 ppm) and f (2.332, 2.344 and 2.356 ppm) corresponding to the alkyl branch hydrogens of the IPFHAs that are shown in Figure 4. These signals were confirmed for the alkyl branch hydrogens of lauric-, myristic- and oleic acids [19,22,23]. Also the signal at g (2.798 ppm) corresponds to hydrogen of CH and the signal at h (1.333 ppm) corresponds to the hydrogens of the methyl groups related to the isopropyl group beside the hydroxamic acid functional group. However ^1^H-NMR signals of hydrogen related to hydroxamic acid group has disappeared due to intermolecular bonding. This phenomenon did not occur in MFHAs.

^1^H-NMR spectra of BFHAs showed the signals at a (0.878, 0.890 and 0.901 ppm), b (1.254, 1.263, 1.295, 1.307 and 1.318 ppm), c (2.009, 2.015, and 2.020 ppm), d (5.337, 5.346, 5.353 and 5.363 ppm), e (1.641 ppm) and f (2.325, 2.338 and 2.357 ppm) corresponding to the alkyl branch hydrogens of the BFHAs that are shown in Figure 5. These signals were confirmed for the alkyl branch hydrogens of, lauric-, myristic- and oleic acids [19,22,23]. Also the signal at g (4.822 ppm) corresponds to the hydrogens of the benzyl group CH_2_. The signal of these hydrogens when located beside the alkyl group appeared in the 2 to 3 ppm region, and when located beside the amine or hydroxylamine group such as in benzyl hydroxylamine, appeared in the 3 to 4 ppm region, hence the appearance of this signal at regions higher than 4 ppm is due to coupling of the acyl groups from oil to benzyl hydroxylamine to form BFHAs. Finally the signals at h (7.322 ppm), i (7.294) and j (7.364) correspond to the hydrogens of the aromatic ring. Similar to IPFHAs, the ^1^H-NMR signals of hydrogen related to hydroxamic acid group had disappeared in BFHAs due to intermolecular bonding.

## 3. Experimental

### 3.1. Materials

*N*-methylhydroxylamine hydrochloride, *N*-isopropylhydroxylamine hydrochloride and *N*-benzyl-hydroxylamine hydrochloride were purchased from Aldrich (USA). Hexane, absolute methanol, hydrochloric acid and sodium hydroxide were supplied by Systerm Co. (Malaysia). Lipozyme TL IM was purchased from Novo Nordisk (Denmark). Commercial palm kernel oil was supplied by Malaysian Palm Oil Board (MPOB, Malaysia).

### 3.2. Synthesis of MFHAs, IPFHAs and BFHAs

MFHAs, IPFHAs and BFHAs were separately synthesized from palm kernel oil according to the method reported recently by our group [19]. Methyl hydroxylaminolysis, isopropyl hydroxylaminolysis and benzyl hydroxylaminolysis separately were carried out by shaking mixtures of the reactants, which contained selected amounts of either *N*-methylhydroxylamine, *N*-isopropylhydroxylamine or *N*-benzyl-hydroxylamine, respectively dissolved in distilled water (10 mL), palm kernel oil (710 mg, 1 mmol) dissolved in hexane (14 mL) and Lipozyme TL IM (80 mg) in a 100 mL flask sealed using Teflon film. The mixtures were shaken at 120 rpm and 39 °C in a water bath shaker for 72 h. The product was separated from the reaction mixture as follow. First the enzyme was filtered. The filtrate was then transferred into a separation funnel for separation of aqueous phase from organic phase. The organic phase in the funnel was shaken with distilled water (10 mL) for removal of residual glycerol, and then 10 mL HCl solution (2 M) was used to remove the unreacted *N*-MHA, *N*-IPHA or *N*-BHA. Hexane was then removed by rotary evaporation to obtain mixture of the product (MPFHAs, PIFHAs or BFHAs) and unreacted oil. Finally the product was extracted from the unreacted oil using absolute methanol (20 mL) and then recovered by rotary evaporation. The percentage of conversion at every experiment was calculated as the following:

Conversion % = A × 100 / B.

A = Amount of experimental fatty hydroxamic acid derivative obtained at every experiment.

B = Amount of theoretical fatty hydroxamic acid derivative: Assuming all of the fatty acids in the oil are converted to fatty hydroxamic acid derivative.

Molecular weight of palm kernel oil = 710 g/mol and its composition is 99% triglyceride (caprylic acid = 1%, capric acid = 3%, lauric acid = 50%, myristic acid = 18%, palmitic acid = 9%, stearic acid = 2%, oleic acid = 15%, linolenic acid = 1%).

### 3.3. Characterization

Qualitative identification of hydroxamic acids were carried out by observing the color of their metal complexes. For this purpose solution of MFHAs, IPFHAs or BFHAs in hexane were mixed separately with 0.01 M of copper (II), iron (III) and vanadium (V) solutions and agitated for about 5 min [18]. The amounts of MFHAs, IPFHAs or BFHAs separately were estimated based on nitrogen content, determined by elemental analyzer (model 932 LECO, USA). Perkin-Elmer 1650 Infrared Fourier Transform Spectrometer was used for recording FTIR spectra. ^1^H nuclear magnetic resonance (^1^H-NMR) spectra were recorded using the NMR Spectrophotometer (Bruker Model AV-III-600, Canada).

## 4. Conclusions

This investigation is the first report which describes the separate synthesis of MFHAs, IPFHAs and BFHAs from the reactions of *N*-MHA, *N*-IPHA and *N*-BHA, respectively, with palm kernel oil, using lipozyme as the catalyst. Among the advantages of these syntheses are the use of an easily available oil and the application of the enzymatic reaction for the purpose of achieving good energy savings for green chemistry purposes. Elemental analysis, ^1^H-NMR and FTIR spectra showed that MFHAs, IPFHAs and BFHAs were produced from *N*-MHA, *N*-IPHA and *N*-BHA, respectively, and the palm kernel oil. The optimum mol ratios of reagents were 6 mmol of *N*-MHA, 7 mmol of *N*-IPHA or 7 mmol of *N*-BHA/mmol of palm kernel oil, while other reaction conditions were: Reaction time, 72 h; shaking rate, 120 rpm; Lipozyme TL IM, 80 mg; hexane, 14 mL; H_2_O, 10 mL; temperature, 39 °C. Finally, by using the optimum conditions the percentage yields of MFHAs, IPFHAs and BFHAs were found to be 75.6, 65.4 and 61.7%, respectively. In summary the MFHAs, IPFHAs and BFHAs are easily prepared at low cost and could be investigated for analytical or biological application in future.
